# Recurrent Spontaneous Pneumothorax in a 42 Years Old Woman With Pulmonary Lymphangioleiomyomatosis: Insights and Pitfalls of the Surgical Treatment

**DOI:** 10.4021/jocmr1170w

**Published:** 2013-01-11

**Authors:** Kyriakos Spiliopoulos, Angeliki Tsantsaridou, Rodula Papamichali, Konstantina Kimpouri, Nicolaos S. Salemis, George K. Koukoulis, Nicolaos B. Tsilimingas

**Affiliations:** a Department of Thoracic and Cardiovascular Surgery, University of Thessaly, Larissa, Greece; b Department of Pathology, University of Thessaly, Larissa, Greece; c Department of Dermatology, University of Athens, A. Syggros Hospital, Athens, Greece; d Breast Surgery Unit, Army General Hospital, Athens, Greece

**Keywords:** Pneumothorax, Lymphangioleiomyomatosis, Renal angiomyolipomas

## Abstract

Lymphangioleiomyomatosis (LAM) is a rare disease that occurs predominantly in females between the ages of 30 and 50 years and is clinically characterized by progressive dyspnoea on exertion, recurrent pneumothoraces, abdominal and thoracic lymphadenopathy, as well tumors-like angiomyolipomas and lymphangiomyomas. We present the case of a 42-year-old woman, who developed recurrent pneumothoraces and was subsequently diagnosed with LAM. Although pneumothorax is a common complication of the disease, its optimal approach to treatment and prevention remains unclear. Chemical or surgical pleurodesis are often performed in order to prevent recurrence, but may predispose to perioperative complications in the event of future lung transplantation.

## Introduction

Lymphangioleiomyomatosis (LAM) is an uncommon, progressive, cystic lung disease that predominantly affects females between the ages of 30 and 50 years. It is sporadic or associated with tuberous sclerosis complex and is characterized by an abnormal proliferation of immature smooth muscle cells (SMC), which grow aberrantly in the airway, parenchyma, lymphatics and pulmonary blood vessels. Clinical manifestations of the disease are progressive dyspnoea on exertion, recurrent pneumothoraces, abdominal and thoracic lymphadenopathy, as well tumors-like angiomyolipomas and lymphangiomyomas. We present here a case of a 42-year old woman, who developed recurrent pneumothoraces and was subsequently diagnosed with LAM. Although pneumothorax is a common complication of the onset, its optimal approach to treatment and prevention remains unclear. The report provides a brief literature review focusing on the surgical management and illustrates insights and pitfalls of these procedures.

## Case Report

A 42-year-old Caucasian Greek woman with a 25 pack-year history of smoking and alcohol abuse was admitted to the hospital complaining of chest pain and progressive dyspnoea on exertion since 4 weeks. Eight years ago, she underwent right nephrectomy due to a giant renal tumor, which was histologically classified as angiomyolipoma.

On examination, the patient was in no acute distress. Her vital signs were normal and her respiratory rate was 14 breaths/min. A head and neck as well a cardiovascular examination were unremarkable. There was no evidence of digital clubbing, lymphadenopathy, oral ulcers or skin lesions. Dullness to percussion and decreased breath sounds were evident over the left lower lung field. No organomegaly, masses or ascites were detectable in the abdomen. There was no clinical evidence of tuberous sclerosis.

Laboratory evaluation revealed a haemoglobin level of 12.9 g/dL and a haematocrit of 40.1%. Her WBC count was within normal range. Serum creatinine was 0.8 mg/dL; urea, 20 mg/dL; glucose, 78 mg/dL; sodium, 133 mEq/L and potassium, 3.9 mEq/L.

**Figure 1:**
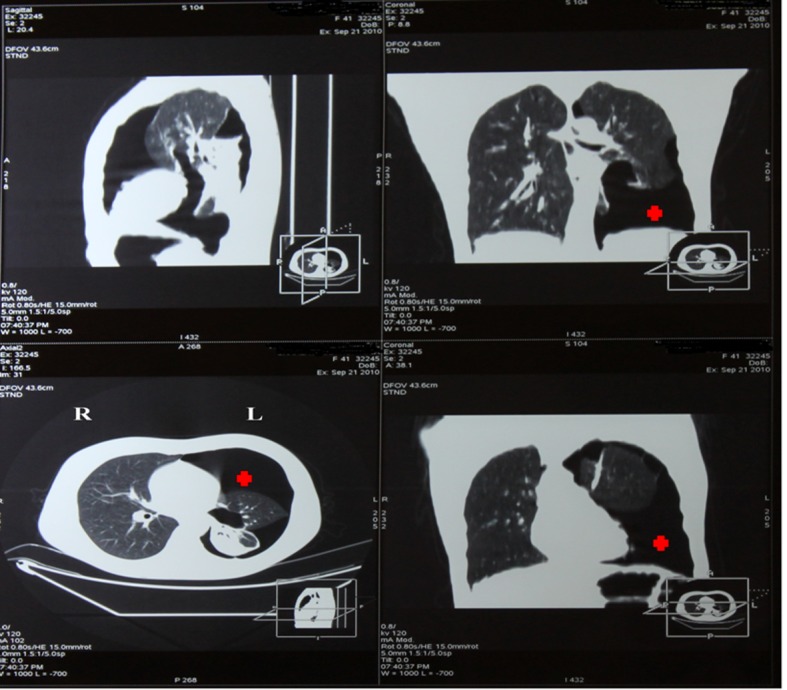
HRCT with big pneumothorax in the left lower hemithorax (red cross).

**Figure 2:**
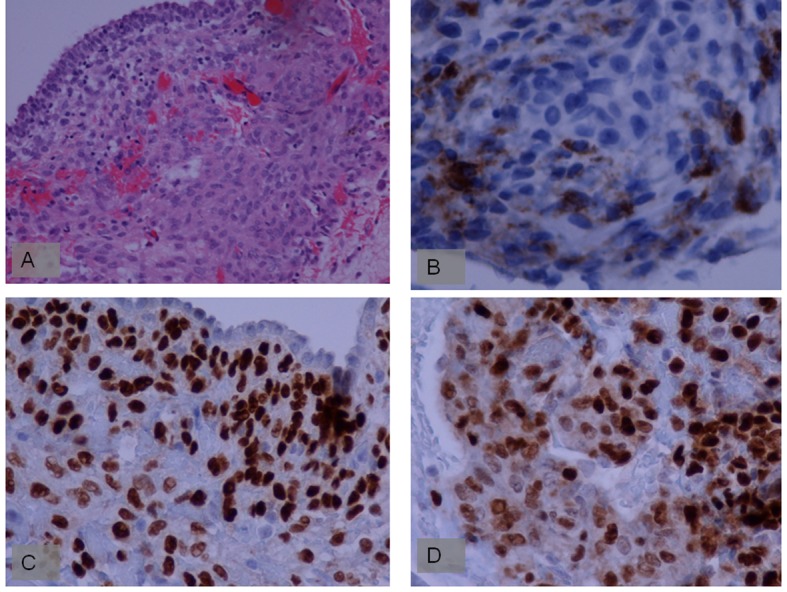
A. Lesional cells are perivascular and consist of polygonal and oval/spindle cells with amphophilic cytoplasm. (H + E, original magnification 200 ×); B. Lesional cells show focal staining with antibody to HMB 45 antigen; C. Lesional cells show staining with antibody to estrogen receptors; D. Lesional cells show staining with antibody to progesterone receptors.

**Figure 3:**
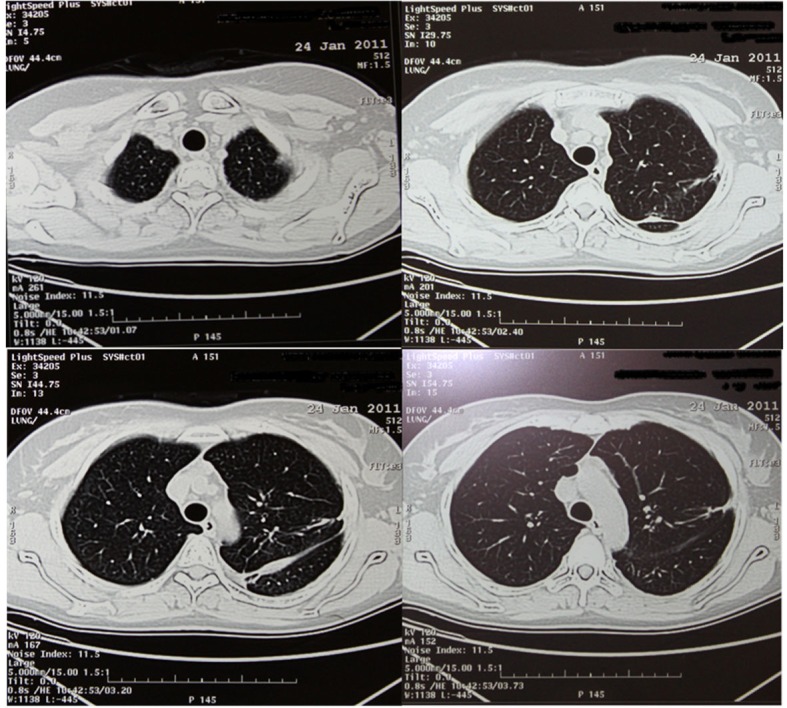
CT scan at follow up (4 months postoperatively).

Chest computed tomography (CT) showed the presence of an extended pneumothorax over the lower lobe of the left lung (Fig. 1). According to the patient’s past medical history, this was the fourth episode (all of them presented on the left side) of pneumothorax within the last two years. The patient refused surgery in the past and was therefore managed conservatively either with thoracostomy tubes and/ or O_2_ supply. Nevertheless after developing the last episode of pneumothorax she accepted surgical management as treatment of choice. We performed a partial small left thoracotomy with resection of two isolated bullae located on the lower lobe and pleurodesis including abrasion of the pulmonary surface and irrigation with 35% dextrose solution. Biopsies of lung tissue showed positive immunoreactivity with monoclonal antibody HMB-45 confirming the diagnosis of LAM (Fig. 2). Follow-up CT scans at 4 months showed a moderate pleural thickening, residual pulmonary cysts on the left side and no evidence of pneumothorax (Fig. 3). On examination the patient was in good physical condition and did not complain of respiratory insufficiency. Regarding concomitant disorders she developed papillary carcinoma of the breast and received therefore radiation therapy.

## Discussion

LAM is a rare disease that occurs predominantly in women of reproductive age. It is characterized by smooth muscle cell infiltration and cystic destruction of the lung [1]. It occurs in 30% of patients with tuberous sclerosis complex (TSC), but patients with LAM complicated by TSC (TSC-LAM) constitute only approximately 15% of all LAM cases that present for medical attention. The remaining cases are sporadic LAM (S-LAM). Renal angiomyolipomas (R-AMLs) occur in about 93% of TSC-LAM patients and about 30-50% of S-LAM cases [1].

Renal AMLs are often mistakenly diagnosed as malignant tumors, causing the immediate removal of the entire kidney, like in the above described case. The main complication of big (> 4 cm in diameter) AMLs is bleeding, which may require blood transfusions. This event is typically characterized by the sudden onset of flank pain with or without the presence of blood in the urine. Under these circumstances, it is indicated either to resect surgically the mass or to perform selective embolization of the tumor [2, 3]. All therapeutic interventions should be performed in a manner to preserve kidney function [2, 3]. Another indication for selective embolization of the tumor may also be severe pain resistant to medical treatment. In cases of large AMLs but without the evidence of bleeding the patients should be followed by an urologist and be referred to an interventional radiologist for eventual treatment. It is still unclear, if prophylactic embolization should be undertaken in these cases. In cases where the diagnosis is difficult to establish, for example in patients with atypical renal masses and without clear evidence of fat and no available information about the growth pattern of the tumor, it is recommended to perform a renal biopsy to rule out malignancy. Those patients with atypical AMLs should be followed by means of CT scans every 6 months, or in order to avoid cumulative radiation exposure, by MRI when available [2]. According to the recently published study of Ryu and coworkers [4] a significant portion of women with sporadic renal AMLs exhibit cystic lung lesions suggestive of pulmonary LAM but may remain undiagnosed. They concluded that the coexistence of pulmonary LAM should be considered in women incidentally found to have sporadic renal AMLs. Therefore the rationale for screening for LAM when an angiomyolipoma is diagnosed, offers a solution in order to identify those patients before developing pulmonary complications.

Pneumothoraces occur in approximately 60-70% of LAM-patients with a recurrence rate of 70%, which is the highest among all chronic lung diseases [1]. They occur either due to direct cyst rupture into the pleural space or indirectly through alveolar wall disruption, followed by leak of air into the lung interstitium, mediastinum, and pleural cavity. Most of LAM-patients with recurrent pneumothoraces will require a pleurodesis procedure. As long as lung transplantation represents the mainstay of therapy for selected patients with advanced pulmonary LAM, some clinicians have advocated postponing definitive preventative measures because pleural interventions are thought to complicate lung transplantation [5, 6]. On the other, data from several studies indicate that conservative approaches (not involving chemical or surgical pleurodesis) to the initial pneumothorax in LAM seldom prevent recurrence and lead to considerable morbidity, strongly suggesting that pleural symphysis should be the goal following the initial event [7].

A total of 80% of patients have their first pneumothorax before the diagnosis of LAM is established and have two or more pneumothoraces before the diagnosis is confirmed [8], which explains why the initial pneumothorax is typically treated conservatively. However, this approach is usually ineffective because of the high recurrence rate.

A study performed by Almoosa et al [8] analyzing among others the efficacy of chemical pleurodesis compared to surgical procedures in the management of pneumothoraces in LAM patients, revealed similar pneumothorax recurrence rates for both treatments (27% vs. 32% respectively). Since surgical interventions are in general associated with a lower secondary spontaneous pneumothorax recurrence rate (about 5%) compared to chemical pleurodesis (approximately 15%) [8, 9], the above reported results underlined the specificity of LAM patients. The reasons for lower success rates after surgical treatment in patients with LAM than to those observed in other diseases [10] are still not clarified. One explanation may be the specific pathology of the LAM lung, characterized by an excessive development of cysts on its surface, which may limit apposition and fusion of the visceral and parietal pleural layers after surgical abrasion of the pulmonary surface [8]. Thus videoasisted thoracoscopic biopsy is the procedure of choice for pleurodesis and lung biopsy in patients with LAM, because it is less invasive than thoracotomy. Resections of bullae are in general not advisable in those patients. Cutting and stapling diffusely diseased lung tissue often result in prolonged leak. Blebectomy may make sense in specific cases, like in our case, where a focal bleb is documented to be leaking at time of thoracotomy. The main concern regarding LAM patients considered for lung transplantation is that past pleural interventions will increase the perioperative bleeding risk and mortality rate. Almoosa and coworkers [8] reported of a 31% perioperative bleeding rate in patients with prior pleurodesis procedures and of those only 50% required rethoracotomy. Their results showed that prior pleural manipulation did not significantly affect the length of hospital stay and did not result in perioperative death. Under the study-limitations that only “survivors” were polled and that there was an unknown percentage of patients, who were rejected for lung transplantation based on a history of pleural procedures, they concluded that prior pleural interventions for recurrent pneumothorax does not preclude successful lung transplantation and that, in general, bleeding complications are manageable.

Innovative surgical approaches like the total pleural covering technique (TPC), which refers to a surgical procedure covering the entire visceral pleura using regenerative oxidized cellulose mesh (ROC) followed by a spray of fibrin glue to coat the ROC, a VATS procedure initially proposed by Kurihara and co-workers [11], or the recently presented modification by Noda et al [12] are very promising and seem to offer safe and reliable solutions for the management of intractable pneumothorax secondary to LAM. TPC accompanied by the use of ROC mesh has a potential advantage to reduce pleural adhesion compared with other pleural interventions, because it causes only a thickening of visceral pleura and does not raise intrathoracic adhesion.

### Conclusion

The diagnosis of LAM should be considered in a woman of any age who presents with R-AMLs. They can be a source of complications and should be followed up carefully by periodic ultrasonography or CT scans in order to preserve kidney function.

Pneumothorax is a common complication of LAM that often recurs prior establishing the definitive diagnosis of the disease. Due to the high recurrence rate, a definitive early surgical intervention should be performed. Although pleurodesis may increase the risk of perioperative bleeding during lung transplantation, this complication is manageable and does not preclude successful transplantation.

The operator’s level of expertise and experience with pleural procedures plays a substantial role regarding the effectiveness of the interventions performed.
